# Editorial: The dynamics of workplace violence: emotions, abusive relationships, and response in organizations

**DOI:** 10.3389/fpsyg.2026.1913769

**Published:** 2026-07-09

**Authors:** Alicia Arenas, Jill Bradley-Geist, Tracy Gonzalez-Padron

**Affiliations:** 1 Department of Social Psychology, University of Seville, Seville, Spain; 2 College of Business, University of Colorado, Colorado Springs, CO, United States

**Keywords:** workplace violence, workplace harassment, workplace abusive behavior, abusive conduct in the workplace, workplace bullying

Workplace violence and harassment represent a critical psychosocial hazard that undermines individual health, organizational functioning, and population wellbeing (Einarsen et al., 2020; International Labour Organization, 2022; Walker, 2025). Defined by the International Labour Organization (ILO) as a range of unacceptable behaviors that result in physical, psychological, or economic harm, these dynamics are increasingly recognized as an organizational rather than merely an interpersonal concern (International Labour Organization, 2022).

Far from being an isolated incident, it is typically characterized by patterns of repeated negative acts where a power imbalance leaves the target vulnerable and unable to easily defend themselves (Einarsen et al., 2003). The research presented in this Research Topic reflects the diverse and often overlapping manifestations of violence in contemporary organizations (Cortina et al., 2001; Edmonson and Zelonka, 2019; Neuman and Baron, 2005):

- **Overt aggression**: including physical assault, verbal abuse, and direct threats.
- **Covert and subtle deviance**: such as co-worker incivility (low-intensity rudeness), negative workplace gossip, and ostracism (intentional social exclusion).
- **Relational and symbolic violence**: manifesting through tyrannical leadership (hostile, humiliating supervisory behaviors) or gender microaggressions—subtle interactional exchanges that convey implicit devaluation.

To fully understand the dynamics of workplace violence, this editorial adopts a multilevel analysis framework (Pais et al., 2026). By synthesizing these perspectives, we move toward a comprehensive understanding of workplace violence as a phenomenon that requires intervention at every level of the organizational system. This Research Topic brings together 12 diverse studies that explore the complex interplay between individual dispositions, relational tensions, and organizational responses to mistreatment.

## The role of individual dispositions and emotions

The role of personality and emotional self-regulation is central to understanding workplace dynamics. In their systematic review and meta-analysis, “*Dark Triad traits and workplace bullying: a systematic review and meta-analysis of personality, power, and psychosocial safety*,” Sui et al. highlight that dark personality traits —Machiavellianism, narcissism, and psychopathy— are robust predictors of bullying perpetration, with psychopathy showing the strongest link to interpersonal aggression.

However, personality is not a fixed risk factor; rather, it interacts dynamically with the environment. As demonstrated by Rosander and Nielsen in “*Being nice, outgoing, curious, organized, and calm—protective or eroded by workplace bullying? Reciprocal effects of personality and bullying, and mechanisms to explain the associations*,” neuroticism has a reciprocal relationship with bullying: it can act as a precursor to mistreatment through interpersonal conflict while also being exacerbated by exposure to bullying through an emotional distress mechanism.

The emotional toll of these experiences often leads to significant clinical and occupational consequences. In “*Tyrannical leadership predicts psychotropic use over time: a longitudinal study of healthcare workers in Chile*,” Ansoleaga et al. show that tyrannical leadership in high-complexity hospitals doubles the odds of workers using psychotropic medications (anxiolytics, antidepressants, or hypnotics) as a coping mechanism for sustained psychological strain. Furthermore, for junior nurses, workplace bullying severely impairs adaptive performance, a relationship mediated by the depletion of cognitive resources and the use of maladaptive emotion regulation strategies like expressive suppression, as explored in “*The relationship between workplace bullying and adaptive performance in junior nurses: the mediating role of emotion regulation and work engagement*” by Du et al.. Finally, a broader view of psychological health in high-stress roles is provided by Ben Salah et al. in “*Crime scene examiners' mental health: a scoping review*”, which identifies a distinct constellation of stressors for forensic staff, including cumulative trauma and professional invisibility.

## Abusive relationships and relational complexity

Workplace mistreatment often manifests through complex relational structures. This Research Topic examines the impact of dual leadership in “*Good cop, bad cop? How (in-)consistencies in dual leadership shape employee strain and commitment*” by Bartz and Felfe. Their study reveals that consistency in positive leadership is the only pattern that fosters commitment and alleviates strain; the positive influence of a transformational leader is effectively neutralized if a second leader in the hierarchy is abusive.

Beyond direct supervisor-subordinate dyads, this Research Topic explores the tripartite relationship involving perpetrators, victims, and third-party witnesses. In “*The contingency effects of abusive supervision of coworker on helping behaviors: the relationships among the perpetrator, the victim, and the third party*,” Sun et al. find that witnesses are less likely to offer help to abused coworkers when they perceive low similarity with the victim or when they have high perceived support from the abusive supervisor. Similarly, covert behaviors are explored in “*Negative workplace gossip and organizational citizenship behavior in the Chinese kindergarten teaching workforce: exploring interpersonal trust as a mediator*” by Xu et al., showing how gossip erodes interpersonal trust, which in turn reduces the willingness of employees to engage in organizational citizenship behavior (OCB). Subtle forms of symbolic violence are further examined by Guillem et al. in “*Measuring workplace gender microaggressions in Spain: validation and measurement invariance of the Spanish version of the MIMI-16*,” which validates a crucial tool for assessing everyday gender-based devaluation that impacts women's occupational health.

## Leadership, organizational, and external regulatory factors

Organizations play a pivotal role in either constraining or enabling violence through their ethical infrastructure. In “*Procedural justice, managerial leadership and gender-based harassment in the workplace: longitudinal cross-lagged associations in a Swedish cohort*,” Peristera et al. demonstrate that procedural justice and supportive leadership serve as long-term antecedents that reduce the risk of gender-based harassment and being a witness to it.

However, this Research Topic identifies a “paradox of moral salience” in “*The paradox of moral salience: when ethical leadership amplifies the impact of coworker incivility on workplace cheating behavior*” by Feng and Chen. They find that leadership can unintentionally amplify the impact of coworker incivility when leaders promote high moral standards but fail to address unchecked peer incivility; the resulting normative discrepancy provides employees with cognitive justifications to morally disengage, potentially leading to unethical behaviors like workplace cheating.

Finally, the Research Topic considers the impact of legal and regulatory shifts in “*Topic modeling of workplace bullying discourse following legal regulation in South Korea*” by Oh et al.. According to their analysis, the introduction of legal frameworks against bullying has caused discursive patterns to become highly procedural. Responses to victimization now predominantly cluster around evidentiary requirements and formal administrative channels, even as the core issue of psychosocial suffering remains an unchanged variable.

## Conclusion

Taken together, the contributions in this Research Topic underscore that addressing workplace violence requires a multilevel prevention strategy. Beyond individual resilience or clear policies, organizations must foster environments that prioritize psychosocial safety, ensure leadership alignment, and recognize the subtle, covert nature of emotional and relational abuse. By unpacking these dynamics, we move toward a more comprehensive understanding of how to protect both the integrity of employees and the health of organizations through countries, professions and organizational contexts.
